# In Vivo Evaluation of the Genotoxic Effects of Poly (Butylene adipate-co-terephthalate)/Polypyrrole with Nanohydroxyapatite Scaffolds for Bone Regeneration

**DOI:** 10.3390/ma12081330

**Published:** 2019-04-24

**Authors:** Conceição de Maria Vaz Elias, Antônio Luiz Martins Maia Filho, Laryssa Roque da Silva, Fabrício Pires de Moura do Amaral, Thomas J. Webster, Fernanda Roberta Marciano, Anderson Oliveira Lobo

**Affiliations:** 1 Biomedical Engineering graduate program, Scientific and Technological Institute, Brasil University, São Paulo, SP 08230-030, Brazil; conceicaovazenf@hotmail.com; 2 Laboratory of Experimental Surgery and Mutagenicity, State University of Piauí, Teresina, PI 64001-280, Brazil; almmaiaf@gmail.com (A.L.M.M.F.); laryssaroqueds@gmail.com (L.R.d.S.); 3 Laboratory of Molecular Biology and Biological Injury Study, State University of Piauí, Teresina, PI 64001-280, Brazil; fabricio34amaral@gmail.com; 4 Department of Chemical Engineering, Northeastern University, Boston, MA 02115, USA; th.webster@neu.edu; 5 Department of Physics, UFPI-Federal University of Piauí, Teresina, PI 64049-550, Brazil; 6 LIMAV-Interdisciplinary Laboratory for Advanced Materials, UFPI-Federal University of Piauí, Teresina, PI 64049-550, Brazil

**Keywords:** Poly (butylene adipate-co-terephthalate), polypyrrole, nanohydroxyapatite, micronucleus, nanotechnology, and comet assays

## Abstract

Here, butylene adipate-co-terephthalate/polypyrrole with nanohydroxyapatite (PBAT/PPy/nHAp) scaffolds were fabricated and characterized. The electrospinning process was carried out using 12 kV, a needle of 23 G, an infusion pump set at 0.3 mL/h, and 10 cm of distance. Afterwards, nHAp was directly electrodeposited onto PBAT/PPy scaffolds using a classical three-electrode apparatus. For in vivo assays (comet assay, acute and chronic micronucleus), 60 male albino Wistar rats with 4 groups were used in each test (n = 5): PBAT/PPy; PBAT/PPy/nHAp; positive control (cyclophosphamide); and the negative control (distilled water). Peripheral blood samples were collected from the animals to perform the comet test after 4 h (for damage) and 24 h (for repair). In the comet test, it was shown that the scaffolds did not induce damage to the % DNA tail and neither for tail length. After the end of 48 h (for acute micronucleus) and 72 h (for chronic micronucleus), bone marrow was collected from each rat to perform the micronucleus test. All of the produced scaffolds did not present genotoxic effects, providing strong evidence for the biological application of PBAT/PPy/nHAp scaffolds.

## 1. Introduction

Currently, around the world, nearly 2.2 million patients are hospitalized for a bone graft procedure to repair bone defects, alleviate suffering, or combat bone diseases every year [1]. Diseases related to bone structures have become a public health problem, posing the greatest challenges for surgical orthopedic reconstruction.

From this, tissue engineering and regenerative medicine have advanced the search for new ways to solve such problems. With an increase in automobile accidents and life expectancy, and consequently an increase in the elderly population, bone fractures and osteoporosis are more common today than ever [2]. Thus, these facts encourage the scientific community to seek new, improved biomaterials for bone replacement or repair.

Bone regeneration is still a complex process and requires the coordination and presentation of biochemical stimuli to promote the multiple stages of angiogenesis and osteogenesis [3]. These processes involve molecular signals mediated primarily by growth factors (GFs) and cytokines. Platelets also contain several GFs and cytokines that play a key role in inflammation and bone repair [4,5].

Tissue engineering is also intended to restore, repair, or aid in the regeneration of impaired tissues and organs for various reasons, using scaffolds, cells, and biomolecules, which together mimic the body’s natural extracellular matrix, providing chemical, physical, and mechanical properties that promote cell adhesion, migration, proliferation, and differentiation [6,7].

Polymeric nanofiber scaffolds simulate the natural nanofibrous nature of bone and have a high surface area and porosity, which are desirable for promoting greater cell density and osteoblast differentiation. These scaffolds act as a temporary framework for cells to regenerate a new extracellular matrix (ECM) [8,9]. The application of biocompatible polymeric nanofibrous scaffolds in orthopedics aims to replace human bone and provide a rapid recovery process through optimization of the bone–implant interface [10].

The nanofibrous scaffold architecture, which can be created by electrospinning, closely resembles the natural environment in which cells reside in the ECM. In this way, the implantation of nanofibrous electrophilic materials has a greater chance of favoring cell adhesion and proliferation, facilitating the process of tissue repair or regeneration. These nanofibers can be obtained from natural or synthetic polymer matrices and their choice depends on the specific therapeutic application. Then, a new material can be produced, which promotes the repair of this tissue, giving it essential characteristics for the correct functioning for the healing of bone tissue [11,12].

Polybutylene adipate co-terephthalate (PBAT), a highly flexible polyester with low crystallinity and good mechanical resistance, has been a good choice for orthopedic tissue engineering, however, it presents low bioactivity [12,13]. In addition, bioceramics derived from calcium phosphates, especially hydroxyapatite (HAp), have a strong chemical similarity with bones and teeth and is highly bioactive [14]. The HAp has the stoichiometric formula, Ca_10_ (PO_4_)_6_(OH)_2_, with a Ca/P ratio of 1.67 and is the least soluble calcium phosphate of all, and the most stable. HAp has been used as a bone replacement material for complete or partial bone augmentation, filling of bones and teeth, or coating of conventional orthopedic and dental implants [15].

Nanohydroxyapatite (nHAp) is a nanoparticle that exhibits chemical and structural similarity to the bone mineral phase and has excellent properties such as biocompatibility, bioactivity, and osteoconductivity, even better properties than conventional or micron structured HAp [16]. Polypyrrole (PPy) is conductive, which can improve bone growth due to its piezoelectric properties. Electrical stimulation can significantly promote the regeneration of bone tissue. Conductive biomaterials can respond to electrical stimuli and, therefore, can mimic the native bone ECM [13,17].

Thus, there has been a major focus on the use of nHAp and conductive materials in bone tissue engineering. However, one major concern about the use of nHAp in orthopedics is that, in theory, nanomaterials (NMs) can induce genotoxic effects by altering the repair of DNA systems in cells or depleting antioxidant defenses. Such events may result in premutagenic lesions that can possibly lead to cancer, among other diseases [18].

In the literature, there are no studies to the best of the authors’ knowledge demonstrating electrodeposition of nHAp on the surface of polyesters with PPy. These scaffolds have been produced only by our research group. The innovation of this material is that nHAp is placed outside the scaffold by electrodeposition, thus providing a bioactive surface on an electrically active material. However, the incorporation of high concentrations of nHAp into polymer solutions necessary for electrodeposition is still a challenge. High concentrations of nHAp in a coating can improve the biocompatibility of the material [15], as well as improve the osteogenesis process. Given this context, the nanofibrous scaffolds used in this study may be relevant for orthopedic regenerative medicine and tissue engineering.

Our group showed for the first time, in a recently published article [19], that PBAT/PPy and PBAT/PPy/nHAp show promise for bone regeneration. In a published article, we showed in vitro tests and, through some characterization tests performed, we found that these scaffolds can stimulate the proliferation and differentiation of human osteoblast MG-63 cells, with no cytotoxic effects. It also showed that the groups of scaffolds containing PPy and nHAp (i.e., differing in the deposition of HA crystals, PBAT/PPy and PBAT/PPy/nHAp) were the two best scaffolds produced; in this work, we will perform complementary tests for later clinical applications. Specifically, here, in vivo tests with micronucleus (MN) and comet assays (being gold standard tests to evaluate genotoxicity and safety) were conducted [19]. Another study has also explored the combination of PBAT with synthetic nHAp (3% and 5% by weight) for bone regeneration. Such in vitro tests showed that PBAT and nHAp were not cytotoxic to MG-63 cells and promoted high cell proliferation rates with the formation of mineralized nodules. From a mechanistic point of view, the loading of nHAp increased hydrophilicity, which, in turn, allowed for a better adsorption of select proteins and consequent alterations in the phenotypic expression of osteoblasts. The scaffold promoted bone formation, presenting the highest bone volume after six weeks of implantation [16]. Another study with PBAT and PPy at 1%, 2%, and 3% showed that the fibers produced could support neuronal differentiation, which is necessary for bone innervation [19].

PBAT/PPy scaffolds with nHAp are still largely unexplored by scholars, and for the first time, this research group investigated the in vivo genotoxic effects of these electrospinning scaffolds [13,16,19], studying the real effect of nanofibers in the cell of living beings. These tests are of great importance, and are regulatory requirements [20] for subsequent clinical applications in humans, in order to evaluate the mutagenic potential of these materials produced in the laboratory for tissue repair.

The guidelines for assessing genotoxicity [20] are crucial in the safety assessment of new materials. The Organization for Economic Co-Operation and Development (OECD) published several guidelines with validated and standardized in vitro and in vivo methods, including genotoxicity tests.

In general, the in vitro or in vivo MN and comet assays are the most frequently used tests to investigate genotoxicity. Thus, there is a need to perform genotoxic tests, such as a comet test and MN test, for this purpose [21,22,23]. However, most articles found in the literature which produce nanofibrous scaffolds perform only in vitro tests to evaluate cytotoxicity, such as the Direct Method Cytotoxicity Test (MTT), saline artemia, and hemolytic activity, which are simplistic tests difficult to translate in vivo.

After obtaining the biomaterial, it is necessary to carry out genotoxic studies of these materials in order to ensure their use and not to bring a future risk of toxicity; this would justify the use of such materials by establishing a strong relationship between exposure to genotoxic agents and the development of several harmful health effects, and by definition, any substance or chemical that damages DNA. The ability of a substance to damage DNA is of concern because this substance may be potentially mutagenic and/or carcinogenic [24].

Here, the following null hypothesis was considered: when there is no significant difference in the increase of the cellular frequencies between the group with scaffolds (PBAT/PPy and PBAT/PPy/nHAp) and the Control Positive (PC), the cyclophosphamide is known as genotoxic [25,26].

The objective of this work was, thus, to produce nanofibrous scaffolds similar to the natural nanofibrous ECM of the bone tissue and to evaluate their in vivo possible genotoxic effects, through the comet and MN tests, as part of the recommended tests for mutagenic potential, in order to guarantee their posterior clinical application for the purpose of regenerating bone tissue.

## 2. Results

**Figure 1:**
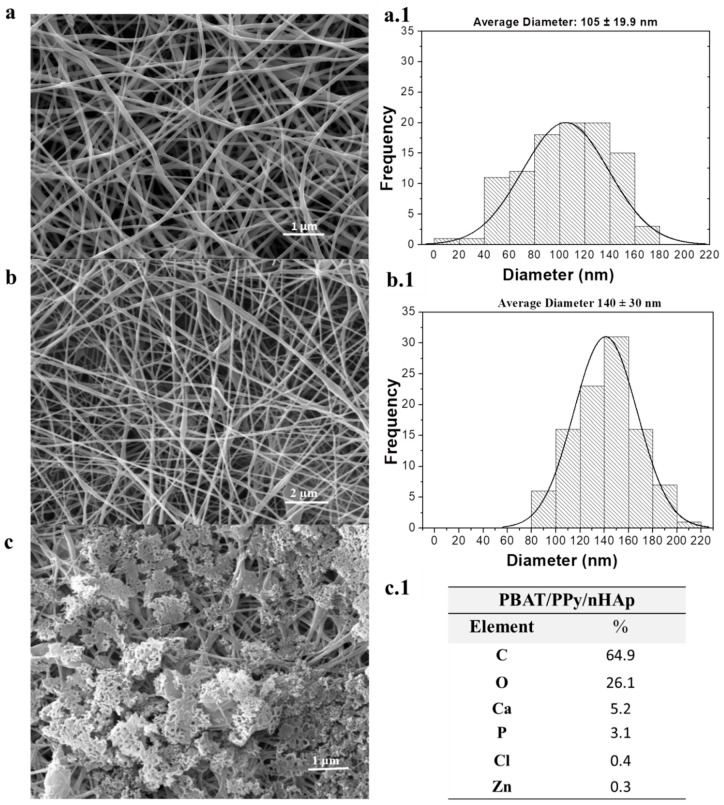
The figure shows SEM micrographs of the scaffolds: (**a**) PBAT, (**b**) PBAT/PPy, (**c**) PBAT/PPy/nHAp; mean diameter of the nanofibers for the PBAT and PBAT/PPy scaffolds as calculated by the Image J program were (**a.1**) 105 ± 19.9 nm and (**b.1**) 140 ± 30 nm, respectively; and the (**c.1**) EDX of the PBAT/PPy/nHAp scaffolds showing its chemistry, presenting a Ca/P ratio of the stoichiometric HAp of 1.67.

Figure 1 shows the micrographs of the nanofibrous scaffolds of PBAT, PBAT/PPy, and PBAT/PPy/nHAp (electrodeposition with nHAp). The scaffolds presented a ‘bead-on-a-string’ morphology. The mean scaffold diameter of the nanofibers, PBAT/PPy (Figure 1b,b.1, 140 ± 30 nm), was larger than that of the PBAT scaffolds (Figure 1a,a.1, 105 ± 19.9 nm), but both were presented as ultrafine fibers. It is important to measure them as nanometer fibers, which have been shown to influence the interaction of the biomaterial with cells [27].

In Figure 1c, nHAp was homogeneously incorporated throughout the PBAT/PPy, thus confirming that the electrospinning conditions used in this work helped in the formation of this biomaterial.

Figure 1c shows the chemical composition of the nanofibrous supports for PBAT/PPY/nHAp (electrodeposited with nHAp), as determined by the coupled X-ray dispersive energy accessory (EDX). After the collection of the EDX spectra, the Ca/P atomic ratio was calculated, where a value equal to 1.67 was determined (Figure 1c,c.1). The EDX (Figure 1c.1) shows the presence of Ca (calcium), P (phosphorus), and O (oxygen). Thus, electrodeposited nHAp crystals were uniformly distributed on the surface of the nanofibrous scaffolds of PBAT/PPy, as shown in the micrographs.

**Figure 2:**
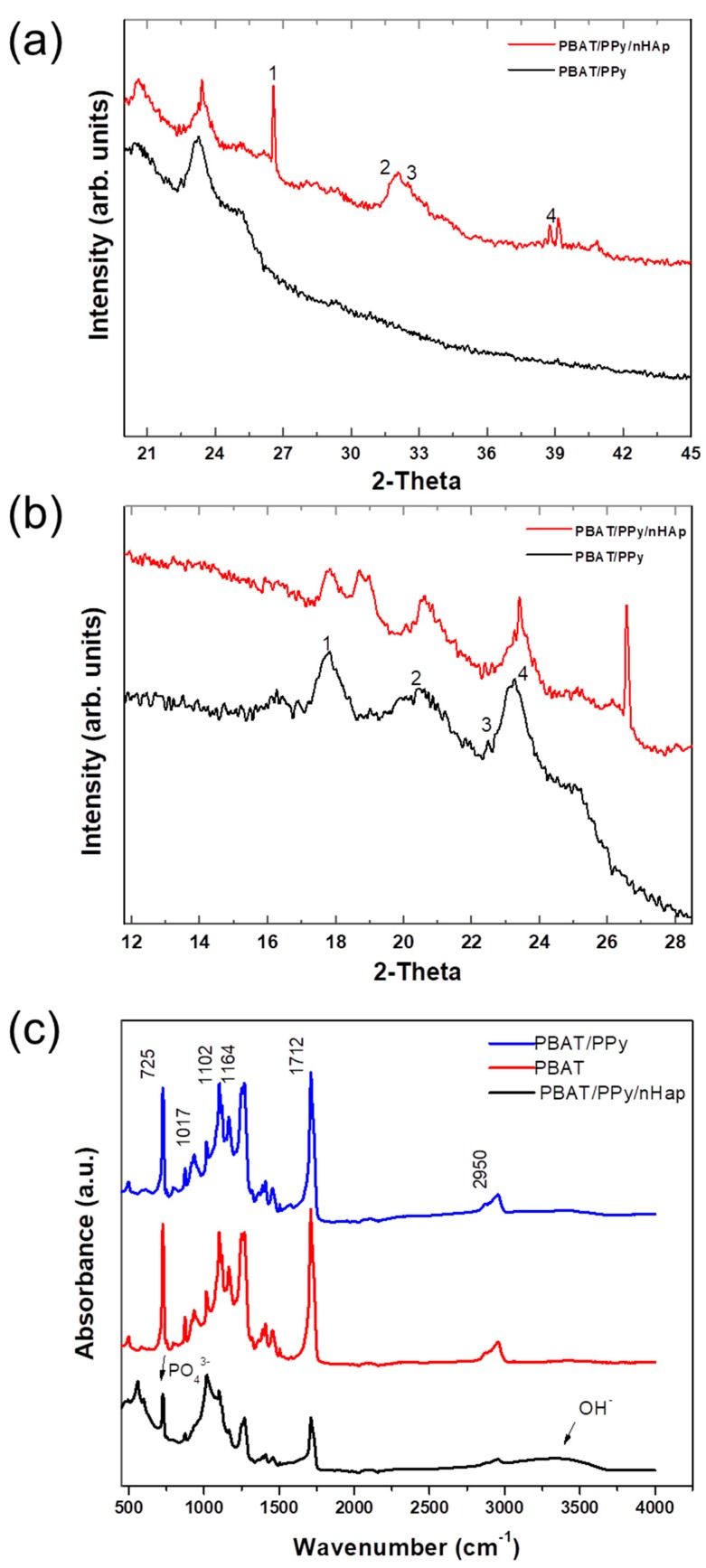
(**a**) X-ray diffraction spectrum of the PBAT/PPy and PBAT/PPy/nHAp scaffolds, with (**b**) a zoom in the 12–30 cm^−1^ region, showing the PBAT polyester. (**c**) ATR-FTIR spectra of the PBAT, PBAT/PPy, and PBAT/PPy/nHAp scaffolds. In figure (**a**), 2θ angles (1) = 26.5°, (2) = 31.9°, (3) = 32.4°, and (4) = 38.7°. In figure (**b**), 2θ angles = (1) = 17.7°, (2) = 20°, (3) = 22.5°, and (4) = 23.3°.

Figure 2a,b shows the XRD patterns of the nanofibrous PBAT/PPy and PBAT/PPy/nHAp scaffolds. The formation of nHAp in the nanofibrous PBAT/PPy scaffolds was confirmed with the appearance of nHAp characteristic peaks, as shown in Figure 2a, at peaks numbered 1 through 4, which correspond to the 2θ angles = 26.5°, 31.9°, 32.4°, and 38.7° with planes (002), (211), (300), and (220), respectively, which are in accordance with JCPDS 01-072-1243. Still in Figure 2b, it shows the PBAT nanofiber peaks numbered from 1 to 4, corresponding respectively to the 2θ angles = 17.7°, 20°, 22.5°, and 23.3°. This confirms that there was effective nHAp deposition and that the PPy did not impair their formation or deposition. The intensity of the XRD peaks for the nanofibrous PBAT/PPy scaffolds increased with the addition of nHAp as shown in Figure 2.

Figure 2c shows the nanofibrous PBAT and PBAT/PPy scaffold FTIR spectra with an asymmetric stretching vibration of CH_2_ groups at 2950 cm^−1^. Another stretch of carbonyl vibration (C = O) is present showing a characteristic of the PBAT polyester at 1712 cm^−1^; symmetric C–O stretching vibration at 1264 cm^−1^ and 1102 cm^−1^; bending of the CH-plane of the phenylene ring at 1017 cm^−1^; and a marked deformation peak of (-CH_2_-) at 725 cm^−1^ of the aromatic compound [28,29].

The electrodeposition of nHAp in nanofibrous PBAT/PPy scaffolds can be confirmed by ATR-FTIR. The vibrational band in the region of 3000 to 3750 cm^−1^ can be attributed to OH-peak absorption, while the characteristic peak absorbance for PO_4_^−3^ could be observed in the band at 600–450 cm^−1^ [30].

Statistical analysis of the data for the evaluation of the genotoxic effects was performed using the GraphPad Prism 6.0 program; a significance level of α = 0.05 was considered for all tests. The results are expressed as means and standard deviation for both the comet and micronucleus tests.

**Figure 3:**
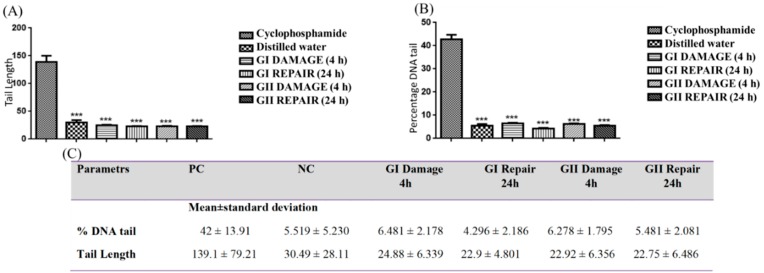
Tail length recorded after analysis of the open comet of the animals after exposure (**A**). Recorded % DNA tail after analysis of the open comet of the animals after exposure (**B**). DNA damage found by group after exposure to PBAT/PPy and PBAT/PPy with nHAp reported as the mean and standard deviation of % DNA tail and tail length (**C**). Legend: NC = negative control; PC = positive control (cyclophosphamide 50 mg/kg). Exposure group: GI = PBAT/PPY/nHAp GII = PBAT/PPy. Results expressed as significance and standard deviation; “***” indicates a statistically significant difference (*p* < 0.001) compared to PC.

For the analysis of the comet test, 100 nucleotides per animal were read, comprising a total of 500 nucleotides per group. Figure 3 illustrates the toxic potential of the PBAT (12%) PPy (1%)/nHAp and PBAT (12%)/PPy (1%) (GI e GII Damage) scaffolds. Note there was a significant difference of % DNA tail of the nanofibrous scaffolds produced (6.481 ± 2.178 and 6.278 ± 1.795), compared to the PC (42.80 ± 13.91) and there was no significant increase in relationship to the NC (5.519 ± 2.178) in the first 4 h and the 24 h groups behaved similarly. This fact was also observed when we analyzed the tail length, suggesting that there was no genotoxic effect.

**Figure 4:**
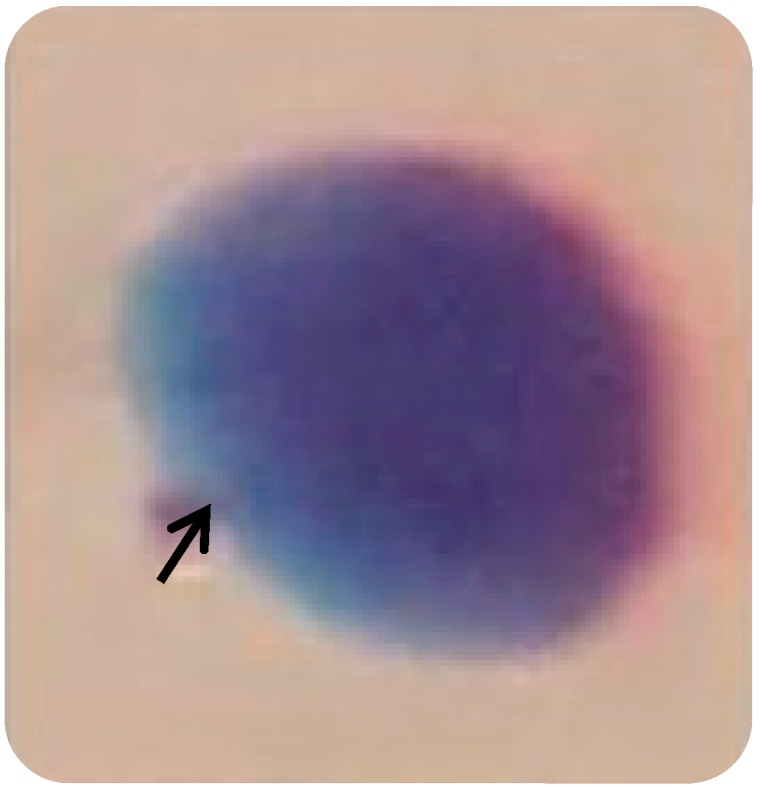
Illustration of the presence of an MN. Image obtained by optical microscopy at 100× showing the presence of an MN by the arrow.

Therefore, the results presented in Figure 3 showed that the membrane did not induce damage in the DNA and genotoxicity of the cells. Figure 4 shows the presence of the micronucleus (MN) in the bone marrow of the Wistar rats (Rattus norvegicus).

The mean and standard deviation of the MN were calculated from the bone marrow cell counts of rats; two sheets were made per animal (n = 5). The rates at different exposure times were evaluated from the bone marrow cells in Wistar rats, being 48 h (for acute) and 72 h (for chronic) of the four groups.

**Figure 5:**
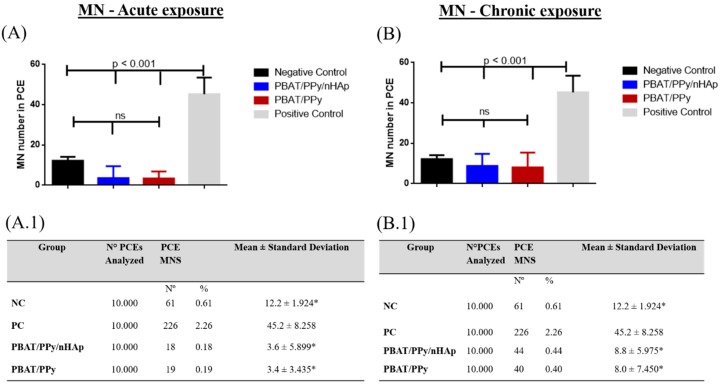
Mean of micronuclei found in 2000 PCEs after acute exposure per animal. (**A**) Mean of micronuclei found in 2000 PCEs after chronic (72 h) exposure per animal. (**B**) Table with frequencies of micronucleated polychromatic erythrocytes (PCEMNs) in Wistar rat bone marrow exposed to the scaffolds. (**A.1**) acute exposure after 48 h. (**B.1**) chronic exposure after 72 h. Legend: NC = negative control; PC = positive control (cyclophosphamide 50 mg/kg); PCEs = polychromatic erythrocytes in the table. * indicates a statistically significant difference (*p* < 0.001), compared to PC.

Figure 5A.1 shows that the PBAT (12%) PPy (1%)/nHAp (Laparotomy + scaffold) scaffold was 3.6 ± 5.899, the PBAT (12%)/PPy (1%) was 3.4 ± 3.435, the PC group (cyclophosphamide) was 45.2 ± 8.258, and the NC group (laparotomy) was 12.2 ± 1.924 for the evaluation of acute micronucleus (48 h).

The MN test was evaluated comparing the frequency of groups found for the different acute exposure times and is reported in Figure 5A, and chronic exposure in Figure 5B. The groups compared were: PBAT/PPy and PBAT/PPy/nHAp, negative control (NC), and positive control (PC), with their respective means and the standard deviations of the MN frequencies for the different exposure times are presented in Figure 5A.1,B.1. As shown in Figure 5, the electrospun fibers before and after electrodeposition of nHAp showed neither acute nor chronic genotoxic damage when analyzed in vivo.

## 3. Discussion

The application of biocompatible polymeric material scaffolds aimed at the replacement of human tissues or organs is increasing in the medical, dental, and tissue engineering fields. For this, it is necessary to understand the interactions between biomaterials and biological tissues for later evaluation of their clinical use [31]. The scaffolds in this study were developed with the purpose of stimulating the regeneration of osseous tissue. In this study, we evaluated their genotoxic potential through the MN and comet test. Some similar results were found in a similar study: using a polystyrene/collagen/norbixin membrane did not present a genotoxic effect when using the comet and MN tests in the bone marrow of rattus novergicus after 72 h [32].

With the characterization carried out in this work, such as FTIR and XRD, it was possible to show with their respective spectra/peaks that the material we proposed to produce was effective. By MEV, we showed the homogeneous formation of nHAp on the surface of the PBAT/PPy supports and that the PPy did not impair the formation of nHAp, and that the nHAp was well distributed. According to Picciani [33], adding a conductive polymer alters the properties of the solution, and hence the formed fibers, by decreasing the formation of the beads and leaving the fibers more uniform (as observed in Figure 1). It was observed that the PBAT and PBAT/PPy nanofibrous scaffolds had a few beads, as shown in Figure 1a,b. According to the literature, polypyrrole is amorphous [34], therefore imperceptible in Figure 2b. All of the peaks were characteristic of the crystalline phase of the respective polymers, as reported in the literature [35]. In addition, with EDX, the Ca/P stoichiometry ratio of the PBAT/PPy/nHAp nanofiber was shown to have chemical similarity to the HAp of bones and teeth (Ca/P = 1.67 [36]). XRD analyses (Figure 2a,b) indexed characteristic peaks of PBAT and PPy which agree with our reported paper [19]. The mixture of these two polymers is important to maintain the mechanical properties and an ability to promote electrical stimuli of the bone cells for the creation of the ideal scaffolds. FTIR analysis (Figure 2c) showed that PBAT/PPy/nHAp scaffold vibration bands were less intense than PBAT/PPy. Thus, the interaction that occurred with the electrodeposition of nHAp on the surface of the scaffolds, reflecting the behavior of the bands, decreased in intensity and vibration [37], and the average diameter of the scaffolds was similar to the natural nanometric structures of the ECM. One of the fundamental aspects in the development of a scaffold is that the 3D structure developed mimics the natural ECM present in the bone tissue. The ECM is primarily composed of a protein known as collagen, which is organized into nanofibers with a diameter of 50 to 500 nm and controls cellular behavior with its architecture. In bone, the basic building block of the ECM is mineralized collagen fibrils. In this way, the importance of nanofibrillar structures for the production of biomimicking scaffolds for the engineering of bone tissues is clear [38].

Since the nanomaterials (NMs) become quite reactive because of their relative increase in surface area, this leaves a larger number of molecules to react with proteins and subsequently cells. The production of NMs has increased considerably in recent decades and today people are exposed to an unknown amount of a wide variety of these materials. The same characteristics that make them interesting for applications in various sectors can also lead to toxicity.

Thus, there is concern about the potential harmful effect of NMs on human health. NMs interact with cells and with their cellular components and some still persist in cells if they are not bioabsorbable. NMs may also enter the nucleus, intentionally or unintentionally, and may interact with the DNA, causing damage as DNA breaks, chromosomal damage, or altered bases. Given the characteristics mentioned, the presence of nHAp has the characteristic of increasing the biocompatibility and bioactivity of new biomaterials, leading to a greater capacity of integration of the NMs into the tissues, promoting and supporting bone growth.

The mixing of these materials is important to maintain essential characteristics to promote bone regeneration, such as improving biocompatibility, mechanical character, electrical stimulation with bone cells, and other benefits that may be obtained when used in clinical practice.

It is important to note that these NMs can reach the nucleus during mitosis and interfere with the microtubules, causing genotoxicity, as the clastogenic effects. They can still alter mitochondrial function causing the production of reactive oxygen and nitrogen species and inducing DNA oxidation. Inflammation produced by NMs in tissues may also affect DNA [18]. Therefore, this more in-depth genotoxicity study is relevant and should be completed after producing any NM.

The comet assay is very useful and widely used for evaluating DNA damage and repair on individual cells. Its basic principle is the lysis of cell membranes. In the microscope, we can identify that the migrated cell takes on the apparent shape of a comet, with a head, nuclear region, and tail, which contains fragments or strands of DNA that migrated toward the anode. The comet analysis is based on the degree of DNA fragmentation and its migration by microelectrophoresis [39].

DNA is an organic molecule responsible for the genetic information of cells. For this information to be transmitted successfully from generation to generation, DNA integrity must be maintained. However, despite its considerable chemical stability, studies on its metabolism have shown the dynamic properties of this molecule [40] and constant exposures to various agents can produce a variety of lesions in their structure. These lesions may affect essential cellular mechanisms such as DNA replication and its transcription into RNA, generating DNA damage that can produce genotoxic (mutagenesis) or cytotoxic (cell death) effects on cells, depending on the nature of these lesions and hence genomic instability and even the onset of cancer [41].

It is important to mention that during the normal metabolism of DNA, there may also be spontaneous changes in its structure that compromise its optimal functioning, such as base mismatch, loss of amino groups present in the bases (cytosine, adenine, and guanine), and so forth. This loss occurs spontaneously in reactions dependent on pH and temperature and if they are altered during their replication, they can generate definitive mutations [42]. Although mutations are essential when one thinks of evolutionary processes, they are first and foremost harmful to biological systems [43].

DNA repair is necessary to maintain the integrity and stability of genomic DNA for normal cell survival, otherwise, the frequency of cell death, gene mutations, or evolution to tumor cells would be increased. Thus, evaluating the DNA situation and its response to damage and repair is a promising strategy to prevent/predict treatment for bone regeneration and to ensure that it does not pose a risk for clinical practice [44].

In a study that conducted a comet assay to assess the genotoxicity of self-renewing ceramics on human lymphocytes cultured on three groups of different ratios of LaPO_4_/Y_2_O_3_ diphase for six days, researchers did not show any difference between the tested ceramics and a negative control (*p* > 0.05), indicating that the biomaterial was safe for clinical trials [45,46].

The comet assay is a quick and inexpensive method for measuring single strand breaks of DNA. It also has an advantage over other DNA damage detection methods such as sister chromatid exchange, alkaline elution, and MN testing because of its high sensitivity [47].

Another study that compared the two techniques of MN and a comet assay to detect the genotoxic effects of X-ray radiation [48] found greater sensitivity for assessing DNA damage for the comet assay than for the MN assay. This underscores the importance of maintaining a combination of tests for better discernment of the mutagenicity of agents. The comet and MN tests are gold standards for the evaluation of genotoxic effects in scaffolds, with the comet test complementary to the micronucleus, corroborating the safety of its use in clinical practice. Micronuclei are small structures that have chromatin juxtaposed to the main nucleus or binucleate daughters after the conclusion of mitosis. These may arise in response to the results of clastogenic, aneuploid, or abnormal chromosomal segregations [49].

When a tissue is exposed to carcinogens, before any clinical symptoms manifests, there is an increased frequency of MN. Thus, the MN test is an occupational biomarker in cells exposed to genotoxic chemical agents, as well as a possible indicator of early carcinogenic signs [50].

It is noted that many works [26] use cyclophosphamide as a positive control (PC), since it is a chemotherapeutic drug belonging to a group of alkylating agents and its main mode of action is based on its interaction with DNA, resulting in repressed cell division, that is, it is genotoxic [25].

In vivo, MN frequency assessment is recommended by international agencies and government institutions to be conducted as part of the battery of tests to assess the safety of a product; it is considered a valuable tool in the assessment of genotoxicity. MN analysis is typically performed on polychromatic erythrocytes (PCE, young erythrocytes which still contain ribosomes) from the bone marrow of mice or rats. The MN is a chromatin mass originating from chromosome fragments or whole chromosomes, which were lost during cell division due to clastogenic (chromosome breakage) or aneugenic events (which induce aneuploidy or abnormal chromosomal segregation). This method, thus, detects chromosome breakage and also abnormal chromosomal segregation [51,52,53].

In summary, the results show that the use of these nanofibrous scaffolds is viable, not inducing aneugenic or clastogenic effects in vivo, suggesting that these biomaterials are promising and safe for numerous applications for tissue engineering. From the MN analyses, we identified significant differences between electrospun scaffolds (PBAT (12%)/PPy (1%) and PBAT (12%) PPy (1%)/nHAp) and PC (*p* < 0.05). Meanwhile, the same electrospun scaffolds did not show statistical differences compared to NC. Through the MN and comet test, it was possible to show that the scaffolds are not genotoxic. These scaffolds present great potential for applications in regenerative medicine as a support for bone growth and are safe to use.

## 4. Materials and Methods

### 4.1. Production of the Solutions

Table 1 summarizes the produced groups. The nanofibrous scaffolds were prepared with the commercial polymer PBAT (Ecoflex^®^ F Blend C1200, BASF, Sao Jose dos Campos, Sao Paulo, Brazil), chloroform (Sigma-Aldrich^®^, St. Louis, MO, USA), as the secondary solvent dimethylformamide (DMF, Sigma-Aldrich^®^, USA) and the secondary polymer and PPy synthesized at the Federal University of the State of São Paulo (Unifesp, Sao Jose dos Campos, Sao Paulo, Brazil). For the electrodeposition of nHAp crystals on PBAT/PPy scaffolds, the following electrolytes were used: 2.5 mM Ca (NO_3_)_2_4H_2_O and 1.5 mM (NH_4_)H_2_PO_4_ (pH = 4.8) (Sigma-Aldrich^®^, St. Louis, USA).

### 4.2. Electrospinning of Nanofibrous Scaffolds PBAT/PPy

The electrospinning of nanofibrous scaffolds containing PBAT 12 wt% and PPy 1 wt%, separately, were prepared using chloroform and dimethylformamide (DMF) as the solvent system (60/40). In a typical preparation, the PBAT pellets were dissolved in chloroform for 120 min under agitation of 250 rpm by means of a magnetic stirrer (Color Squid IKAMAG^®^, Staufen, Germany) and a magnetic bar until the granules dispersed completely. The PPy was dispersed in DMF under sonication (VCX 500-Sonics, Newtown, PA, USA) for 10 min. After the PPy was completely dispersed, the two solutions were mixed and the resulting solution was stirred for 20 h until complete homogenization occurred.

The polymer solutions were electrospun in a suitable low-cost system using a BERTAN 230-30R high voltage source. The solution was placed in an infusion pump in a 3 mL syringe (BectonDicKinson-BD, Franklin Lakes, NJ, USA) with a disposable needle (23 G) with an inner diameter of 0.33 mm and an outer diameter of 0.63 mm, and with regulated flow at 0.3 mL/h. The process occurred at a distance of 12 cm and an applied voltage of 12 kV (PBAT/PPy) was used. The relative air humidity was controlled, maintaining around 45% to 50%. The samples were collected on a flat steel electrode of 92 × 78 mm dimensions, covered by aluminum foil. The process for each scaffold lasted approximately 1 h per sample.

### 4.3. Electrodeposition of nHAp on Nanofibrous Scaffolds of PBAT/PPy

The electrodeposition of the nHAp crystals on the PBAT (12%)/PPy(1%) nanofibrous scaffolds was performed using the following electrolytes, 2.5 mM Ca(NO_3_)_2_4H_2_O, and 1.5 mM (NH_4_)H_2_PO_4_ (pH = 4.8) (Sigma-Aldrich^®^, St. Louis, USA). These concentrations were chosen according to Ca/P = 1.67. The nanofibrous scaffold PBAT/PPy blends were used as an anode coupled to an electrode holder with an exposed circular area of 2.8 × 10^−5^ m^2^. A platinum rod 5 × 10^−2^ m long and 2 × 10^−4^ m in diameter was used as the counter electrode.

The electrochemical deposition process was performed using a potentiostat/galvanostat (AUTOLAB, PGSTAT 128N, HOLANDA), operating in potentiostat mode, at a constant potential of −3.8 V for 1800 s. To maintain the solution at 70° using a thermostatic bath (QUIMIS, model Q-218-1, São Paulo, Brazil) was used. The entire process was carried out with the aid of a magnetic stirrer (IKA^®^ C-MAG HS4, Staufen, Germany) under continuous stirring of 450 rpm.

### 4.4. Morphological and Structural Characterization of the Scaffolds

The surface morphology of the samples, as well as the layout, shape, and determination of the mean diameters of the nanofibers, were obtained using a Scanning Electron Microscope (SEM) of Zeiss brand, model EVO MA10 coupled to Energy Dispersive X-ray Spectrometry (EDS) for chemical assessment. A thin layer of gold (−10 nm) was deposited on each sample by a sputtering technique, under Argon plasma, at 2 × 10^−1^ mbar, and a current of 30 mA, for 2 min. The average diameter of the scaffold nanofibers was measured using Image J.

Fourier Transform Infrared Spectroscopy (FTIR) was also used with the aid of a Total Attenuated Reflectance (ATR) accessory using a Perkin-Elmer Spotlight 400 FTIR Imaging System. Data were collected in the range of 4000–450 cm^−1^ in absorbance mode.

X-ray diffraction (XRD, X-Pert Philips, Almelo, Netherland) with Cu K-α radiation generated at 40 kV and 50 mA was further used to characterize the microstructure and phase content of the nHAp crystals. The results were compared to a HAp standard (JCPDS pattern 01-072-1243).

### 4.5. In Vivo Tests

#### 4.5.1. Animals

The rats (Wistar, males, adults, 100–120 g) were obtained from the State University of Piauí (UESPI, Teresina, Piaui, Brazil) and all in vivo procedures were approved by a research ethical committee protocol (0029/2017, 20 March 2017). For in vivo experiments, 5 rats were kept in cages with food and water ad libitum and under a light/dark cycle (12 h). A total of 60 Wistar rats were used to verify genotoxic effects, with 20 animals per test: comet, acute, and chronic MN.

#### 4.5.2. Comet Tests

The test was performed with 20 animals, four groups with five animals (n = 5) in each group: (1) the nanofibrous support PBAT (12%)/PPy (1%) and (2) PBAT (12%) PPy (1%)/nHAp, sized to 1 × 1 cm^2^ and introduced into the peritoneum by a laparotomy; (3) the PC group, which received cyclophosphamide known to be genotoxic [25,26], at a dose of 50 mg/kg intraperitoneally; and (4) NC, using intraperitoneally distilled water, in the same proportion as PC. For the experiment, each animal was fully anesthetized with ketamine (1.0 mL/kg) and xylazine (1.1 mL/kg) intramuscularly.

Samples were processed 4 h after exposure (to observe the damage) and 24 h after treatment (to observe the repair). At the end of each exposure (4 h and 24 h), blood samples were collected from the animal’s tail and 40 μL of this sample was transferred to microtubes containing 120 mL of low-melting-point agarose (1.5%) at 37 °C. The mixture was homogenized and transferred to precoated slides with 5% agarose.

The slides were then covered with coverslips and placed at 30 °C for 20 min [54]. The coverslips were removed, and the slides were immersed in a lysis solution with 89 mL of a concentrated solution (of 2.5 mM NaCl, 100 mM EDTA, 10 mM Tris, pH = 10.0, adjusted with solid NaOH, 890 mL distilled water, and 1% sodium laurylsarcosinate), 1 mL Triton X-100, and 10 mL DMSO for 1 h at 3 °C in the dark.

For DNA denaturation, the slides were placed in an electrophoresis cell containing a buffer solution (300 mM NaOH and 1 mM EDTA, prepared from a stock solution of 10 N NaOH and 200 mM EDTA, pH = 10.0) pH > 13 to 3 °C for 20 min in the dark. Electrophoresis was performed at 25 V and 300 mA. The slides were then neutralized using 0.4 M Tris-HCL, pH = 7.5, for 3 cycles of 5 min each.

After this whole process, the slides were then washed with distilled water and air dried, washing them with Gel Red (2.0 Dilution: 10,000 μL for 10 min). At the end of the experiment, the animals were euthanized by cervical dislocation.

All slides were analyzed by immunofluorescence microscopy (40× magnification) equipped with an excitation filter (420–490 nm) and a barrier filter (520 nm). Images were obtained by the Opton System (5.0 megapixel CCD digital camera for immunofluorescence). Thus, the DNA damage was evaluated by the percentage of DNA measurement in the tail (% DNA-measurement of the proportion of the total DNA present in the tail) and the time of the tail (Tail Length-TM times the percentage of DNA in the tail) [55]. These parameters were calculated for 100 nucleotides per sample, two slides per specimen. For this, we used OpenComet software (Cambridge, MA, USA) [56]. Statistical analysis was performed by ANOVA followed by a Tukey’s test (*p* < 0.05).

#### 4.5.3. Micronucleus Tests

The test was performed with 40 animals, 20 Wistar rats for acute and 20 for chronic exposure. Each test was performed with four groups with five animals (n = 5) in each group. The scaffolds were implanted intraperitoneally from the animal by a laparotomy, where the sample size was 1 × 1 cm^2^. The groups tested were: PBAT (12%)/PPy (1%) and PBAT (12%)/PPy (1%)/nHAp; PC, which received cyclophosphamide, known to be genotoxic and to induce DNA damage to bone marrow cells at the dose of 50 mg/kg; and the NC, for which distilled water was used.

All animals were fully anesthetized with ketamine (1.0 mL/kg) and xylazine (1.1 mL/kg) intramuscularly to receive each exposure. The animals were euthanized after 48 h (acute exposure) and 72 h (chronic exposure) after the beginning of the experiments.

The work follows the standards of OECD-474, 2013, where the samples for initial analysis cannot be before 24 h of treatment, and do not extend beyond 48 h after treatment with interval(s) between samples, if the use of sampling times before 24 h after treatment should be justified and should have appropriate intervals after the first sample, but not extending beyond 72 h [20].

To prepare the slide, bone marrow cells were collected immediately after euthanasia, performed by cervical dislocation of the animals. A 1 mL syringe was used for collection. This syringe was filled with fetal bovine serum (FBS). The needle was inserted into the opening of one end of the femur and the fetal serum was flushed through the channel, pushing the spinal component toward the other end, where the Falcon tube, previously marked with the animal code, was located. The scaffolds from the bone marrow were resuspended in fetal bovine serum until homogeneity was achieved.

The suspension was centrifuged for 5 min at 1000 rpm, discarding the supernatant at the end of the Pasteur Pipette procedure. The sample was filled with 0.5 mL of fetal bovine serum and suspended until homogenization. The smears were prepared by dripping 2 drops of the suspension onto the matte end of a slide (previously marked with the animal’s code) and, with the aid of another slide inclined at a 45-degree angle, the smear was made. After the smear was made, the slides were dried in the open air; 2 slides per animal were made. The coloration took 24 h after the blades were made in Giemsa for 3 min.

The blade analysis was performed in a blind field with an increase of 100× (objective of immersion) over a short time by the same observer. The frequency of MN was measured in 2.000 PCE cells per animal, totaling 10.000 PCE/group. The entire protocol was based on numerous publications [57,58,59,60,61].

#### 4.5.4. Statistical Analysis

Data were analyzed using One-Way ANOVA and a Tukey test. A significance level of 5% was considered for all tests. The results are expressed as the means with the standard deviation of the two independent experiments. The result was considered positive when there was a statistically significant increase (*p* < 0.05).

## 5. Conclusions

It was possible to produce nanofibrous scaffolds of PBAT/PPy/nHAp, which presented a similar length to the nanostructures of the natural extracellular matrix and the electrodeposition technique allowed for the formation of a homogeneous nHAp film on the surface. To date, few studies have evaluated the in vivo genotoxic effects of nanofibrous scaffolds. Herein, the MN and comet assays showed that the produced nanofibrous scaffolds were not genotoxic and could be safely used as scaffolds in clinical practice. Therefore, this biomaterial has great potential for applications in regenerative medicine as a support for bone growth.

## Figures and Tables

**Table 1:** Groups of nanofibrous scaffolds, percentage of PBAT, PPy, presence of nHAp, voltage, and flow rate.

Nº	Groups of Nanofibrous Scaffolds	PBAT (wt)	PPy (wt)	nHAp	Voltage (kV)	Flow Rate (mL/h)
1	PBAT (12%)	12%	0	No	12–13 kV	0.3
2	PBAT (12%)/PPy (1%)	12%	1%	No	12–13 kV	0.3
3	PBAT (12%) PPy (1%)/nHAp	12%	1%	Yes	12–13 kV	0.3

## References

[1] Van der Stok J., Van Lieshout E.M.M., El-Massoudi Y., Van Kralingen G.H., Patka P. (2011). Bone substitutes in the Netherlands—A systematic literature review. Acta Biomater..

[2] Mendes L.S., Saska S., Martines M.A.U., Marchetto R. (2013). Nanostructured materials based on mesoporous silica and mesoporous silica/apatite as osteogenic growth peptide carriers. Mater. Sci. Eng. C.

[3] Bayer E.A., Gottardi R., Fedorchak M.V., Little S.R. (2015). The scope and sequence of growth factor delivery for vascularized bone tissue regeneration. J. Control. Release.

[4] Malhotra A., Pelletier M.H., Yu Y., Walsh W.R. (2013). Can platelet-rich plasma (PRP) improve bone healing? A comparison between the theory and experimental outcomes. Arch. Orthop. Trauma Surg..

[5] Intini G. (2009). The use of platelet-rich plasma in bone reconstruction therapy. Biomaterials.

[6] Hinderer S., Schesny M., Bayrak A., Ibold B., Hampel M., Walles T., Stock U.A., Seifert M., Schenke-Layland K. (2012). Engineering of fibrillar decorin matrices for a tissue-engineered trachea. Biomaterials.

[7] Gautam S., Dinda A.K., Mishra N.C. (2013). Fabrication and characterization of PCL/gelatin composite nanofibrous scaffold for tissue engineering applications by electrospinning method. Mater. Sci. Eng. C Mater. Biol. Appl..

[8] Agarwal S., Wendorff J.H., Greiner A. (2009). Progress in the field of electrospinning for tissue engineering applications. Adv. Mater..

[9] Chandrasekaran A.R., Venugopal J., Sundarrajan S., Ramakrishna S. (2011). Fabrication of a nanofibrous scaffold with improved bioactivity for culture of human dermal fibroblasts for skin regeneration. Biomed. Mater..

[10] Costa C., Teixeira J.P. (2012). Efeitos genotóxicos dos pesticidas. Revis. Ciênc. Agrár..

[11] Ingavle G.C., Leach J.K. (2014). Advancements in electrospinning of polymeric nanofibrous scaffolds for tissue engineering. Tissue Eng. Part B Rev..

[12] Khorshidi S., Solouk A., Mirzadeh H., Mazinani S., Lagaron J.M., Sharifi S., Ramakrishna S. (2016). A review of key challenges of electrospun scaffolds for tissue-engineering applications. J. Tissue Eng. Regen. Med..

[13] Ribeiro Neto W.A., de Paula A.C.C., Martins T.M.M., Goes A.M., Averous L., Schlatter G., Suman Bretas R.E. (2015). Poly (butylene adipate-co-terephthalate)/hydroxyapatite composite structures for bone tissue recovery. Polym. Degrad. Stab..

[14] Fihri A., Len C., Varma R.S., Solhy A. (2017). Hydroxyapatite: A review of syntheses, structure and applications in heterogeneous catalysis. Coord. Chem. Rev..

[15] Szczes A., Holysz L., Chibowski E. (2017). Synthesis of hydroxyapatite for biomedical applications. Adv. Coll. Interf. Sci..

[16] Santana-Melo G.F., Rodrigues B.V.M., da Silva E., Ricci R., Marciano F.R., Webster T.J., Vasconcellos L.M.R., Lobo A.O. (2017). Electrospun ultrathin PBAT/nHAp fibers influenced the in vitro and in vivo osteogenesis and improved the mechanical properties of neoformed bone. Coll. Surf. B Biointerf..

[17] Gajendiran M., Choi J., Kim S.-J., Kim K., Shin H., Koo H.-J., Kim K. (2017). Conductive biomaterials for tissue engineering applications. J. Ind. Eng. Chem..

[18] Azqueta A., Dusinska M. (2015). The use of the comet assay for the evaluation of the genotoxicity of nanomaterials. Front. Genet..

[19] De Castro J.G., Rodrigues B.V.M., Ricci R., Costa M.M., Ribeiro A.F.C., Marciano F.R., Lobo A.O. (2016). Designing a novel nanocomposite for bone tissue engineering using electrospun conductive PBAT/polypyrrole as a scaffold to direct nanohydroxyapatite electrodeposition. RSC Adv..

[20] OECD (Organisation for Economic Cooperation and Development) (1997). Guideline 474 (2014) OECD Guidelines for Testing of Chemicals: Mammalian Erythrocyte Micronucleus Test.

[21] Singh N., Manshian B., Jenkins G.J., Griffiths S.M., Williams P.M., Maffeis T.G., Wright C.J., Doak S.H. (2009). NanoGenotoxicology: The DNA damaging potential of engineered nanomaterials. Biomaterials.

[22] Landsiedel R., Kapp M.D., Schulz M., Wiench K., Oesch F. (2009). Genotoxicity investigations on nanomaterials: Methods, preparation and characterization of test material, potential artifacts and limitations—Many questions, some answers. Mutat. Res..

[23] Arora S., Rajwade J.M., Paknikar K.M. (2012). Nanotoxicology and in vitro studies: The need of the hour. Toxicol. Appl. Pharmacol..

[24] Márquez Fernández M.E., López Ortiz J.B., Correa Londoño G., Pareja López A., Giraldo Solano N.A. (2003). Detección del daño genotóxico agudo y crónico en una población de laboratoristas ocupacionalmente expuestos. Iatreia.

[25] Besse J.P., Latour J.F., Garric J. (2012). Anticancer drugs in surface waters: What can we say about the occurrence and environmental significance of cytotoxic, cytostatic and endocrine therapy drugs?. Environ. Int..

[26] Kour J., Ali M.N., Ganaie H.A., Tabassum N. (2017). Amelioration of the cyclophosphamide induced genotoxic damage in mice by the ethanolic extract of Equisetum arvense. Toxicol. Rep..

[27] Wang S., Zhong S., Lim C.T., Nie H. (2015). Effects of fiber alignment on stem cells–fibrous scaffold interactions. J. Mater. Chem. B.

[28] Cai Y., Lv J., Feng J. (2013). Spectral Characterization of Four Kinds of Biodegradable Plastics: Poly (Lactic Acid), Poly (Butylenes Adipate-Co-Terephthalate), Poly (Hydroxybutyrate-Co-Hydroxyvalerate) and Poly (Butylenes Succinate) with FTIR and Raman Spectroscopy. J. Polym. Environ..

[29] Bheemaneni G., Saravana S., Kandaswamy R. (2018). Processing and Characterization of Poly (butylene adipate-co-terephthalate)/Wollastonite Biocomposites for Medical Applications. Mater. Today Proc..

[30] Wang X., Song G., Lou T. (2010). Fabrication and characterization of nano-composite scaffold of PLLA/silane modified hydroxyapatite. Med. Eng. Phys..

[31] Pagano S., Chieruzzi M., Balloni S., Lombardo G., Torre L., Bodo M., Cianetti S., Marinucci L. (2019). Biological, thermal and mechanical characterization of modified glass ionomer cements: The role of nanohydroxyapatite, ciprofloxacin and zinc l-carnosine. Mater. Sci. Eng. C Mater. Biol. Appl..

[32] Miranda Monte S., Filho A., Amaral F., Cabral Leão Ferreira D., Matias Nascimento W., Galber Freitas Viana V., Lúcia Oliveira Monte Z., Costa C., Moura-Neto V. (2016). Genotoxicity Evaluation of Polystyrene Membrane with Collagen and Norbixin by Micronucleus Test and Comet Assay. J. Pharm. Sci..

[33] Picciani P.H.S., Medeiros E.S., Pan Z., Wood D.F., Orts W.J., Mattoso L.H.C., Soares B.G. (2010). Structural, Electrical, Mechanical, and Thermal Properties of Electrospun Poly(lactic acid)/Polyaniline Blend Fibers. Macromol. Mater. Eng..

[34] Campos R.A.M., Faez R., Rezende M.C. (2014). Síntese do polipirrol com surfactantes aniônicos visando aplicações como absorvedores de micro-ondas. Polímeros.

[35] Santos R.A.L., Muller C.M.O., Grossmann M.V.E., Mali S., Yamashita F. (2014). Starch/poly (butylene adipate-co-terephthalate)/montmorillonite films produced by blow extrusion. Quím. Nova.

[36] Grinet M.A.V.M., Zanin H., Campos Granato A.E., Porcionatto M., Marciano F.R., Lobo A.O. (2014). Fast preparation of free-standing nanohydroxyapatite–vertically aligned carbon nanotube scaffolds. J. Mater. Chem. B.

[37] Chaudhuri B., Mondal B., Ray S.K., Sarkar S.C. (2016). A novel biocompatible conducting polyvinyl alcohol (PVA)-polyvinylpyrrolidone (PVP)-hydroxyapatite (HAP) composite scaffolds for probable biological application. Coll. Surf. B Biointerf..

[38] Que R.A., Chan S.W.P., Jabaiah A.M., Lathrop R.H., Da Silva N.A., Wang S.-W. (2015). Tuning cellular response by modular design of bioactive domains in collagen. Biomaterials.

[39] Brianezi G., Camargo J.L.V.d., Miot H.A. (2009). Desenvolvimento e validação de técnica quantitativa de análise de imagem para avaliação do teste do cometa corado pela prata. J. Bras. Patol. Med. Laborat..

[40] Friedberg E.C. (2003). DNA damage and repair. Nature.

[41] De Boer J., Hoeijmakers J.H. (2000). Nucleotide excision repair and human syndromes. Carcinogenesis.

[42] Hoeijmakers J.H. (2001). Genome maintenance mechanisms for preventing cancer. Nature.

[43] Friedberg E.C., Walker G.C., Siede W., Wood R.D., Schultz R.A., Ellenberger T. (2005). DNA Repair and Mutagenesis.

[44] Portich J.P., dos Santos R.P., Kersting N., Jorge K.B., Casagrande P.R., dos Santos Costa G., Dias Cionek J.M.G., Olguins D.B., Sinigaglia M., Busatto F.F. (2017). DNA damage response in patients with pediatric Acute Lymphoid Leukemia during induction therapy. Leukemia Res..

[45] Hua N., Zhu H., Zhuang J., Chen L. (2014). Genotoxicity test of self-renovated ceramics in primary human peripheral lymphocytes. Cell Biochem. Biophys..

[46] Chen S.-J., Bai Y., Huang X.-B., Suo J.-P., Li J. (2012). Genotoxic and Biological Evaluation of a Nano Silica Cross Linked Composite Specifically Used for Intra-Vas Device. Soft Nanosci. Lett..

[47] Lee R.F., Steinert S. (2003). Use of the single cell gel electrophoresis/comet assay for detecting DNA damage in aquatic (marine and freshwater) animals. Mutat. Res..

[48] He J.L., Chen W.L., Jin L.F., Jin H.Y. (2000). Comparative evaluation of the in vitro micronucleus test and the comet assay for the detection of genotoxic effects of X-ray radiation. Mutat. Res..

[49] Albas C.S., Souza J.P., Nai G.A., Parizi J.L.S. (2014). Avaliação da genotoxicidade da Ilex paraguariensis (erva mate) pelo teste do micronúcleo. Rev. Bras. Plantas Med..

[50] Singaraju M., Singaraju S., Parwani R., Wanjari S. (2012). Cytogenetic biomonitoring in petrol station attendants: A micronucleus study. J. Cytol..

[51] Krishna G., Hayashi M. (2000). In vivo rodent micronucleus assay: Protocol, conduct and data interpretation. Mutat. Res..

[52] Iarmarcovai G., Bonassi S., Botta A., Baan R.A., Orsiere T. (2008). Genetic polymorphisms and micronucleus formation: A review of the literature. Mutat. Res..

[53] Serrano-Garcia L., Montero-Montoya R. (2001). Micronuclei and chromatid buds are the result of related genotoxic events. Environ. Mol. Mutagenesis.

[54] Da Silva C.J., dos Santos J.E., Satie Takahashi C. (2010). An evaluation of the genotoxic and cytotoxic effects of the anti-obesity drugs sibutramine and fenproporex. Hum. Exp. Toxicol..

[55] Kumaravel T.S., Vilhar B., Faux S.P., Jha A.N. (2009). Comet Assay measurements: A perspective. Cell Biol. Toxicol..

[56] Gyori B.M., Venkatachalam G., Thiagarajan P.S., Hsu D., Clement M.V. (2014). OpenComet: An automated tool for comet assay image analysis. Redox Biol..

[57] Hayashi M., Morita T., Kodama Y., Sofuni T., Ishidate M. (1990). The micronucleus assay with mouse peripheral blood reticulocytes using acridine orange-coated slides. Mutat. Res..

[58] Hayashi M., Tice R.R., MacGregor J.T., Anderson D., Blakey D.H., Kirsh-Volders M., Oleson F.B., Pacchierotti F., Romagna F., Shimada H. (1994). In vivo rodent erythrocyte micronucleus assay. Mutat. Res..

[59] Hamada S., Ohyama W., Takashima R., Shimada K., Matsumoto K., Kawakami S., Uno F., Sui H., Shimada Y., Imamura T. (2015). Evaluation of the repeated-dose liver and gastrointestinal tract micronucleus assays with 22 chemicals using young adult rats: Summary of the collaborative study by the Collaborative Study Group for the Micronucleus Test (CSGMT)/The Japanese Environmental Mutagen Society (JEMS)—Mammalian Mutagenicity Study Group (MMS). Mutat. Res. Genet. Toxicol. Environ. Mutagenesis.

[60] Benigni R., Bossa C., Tcheremenskaia O., Battistelli C.L., Crettaz P. (2012). The new ISSMIC database on in vivo micronucleus and its role in assessing genotoxicity testing strategies. Mutagenesis.

[61] Kang S.H., Kwon J.Y., Lee J.K., Seo Y.R. (2013). Recent advances in in vivo genotoxicity testing: Prediction of carcinogenic potential using comet and micronucleus assay in animal models. J. Cancer Prev..

